# Supplementary material for: Identification of Key Active Constituents in Eucommia ulmoides Oliv. Leaves Against Parkinson’s Disease and the Alleviative Effects via 4E-BP1 Up-Regulation
Source: Int J Mol Sci. 2025 Mar 19;26(6):2762. doi: 10.3390/ijms26062762 (PMC11943294; doi:10.3390/ijms26062762)
Supplement: Supplementary file 1 [file ijms-26-02762-s001.zip › Figure S1.pptx]

## Slide 1
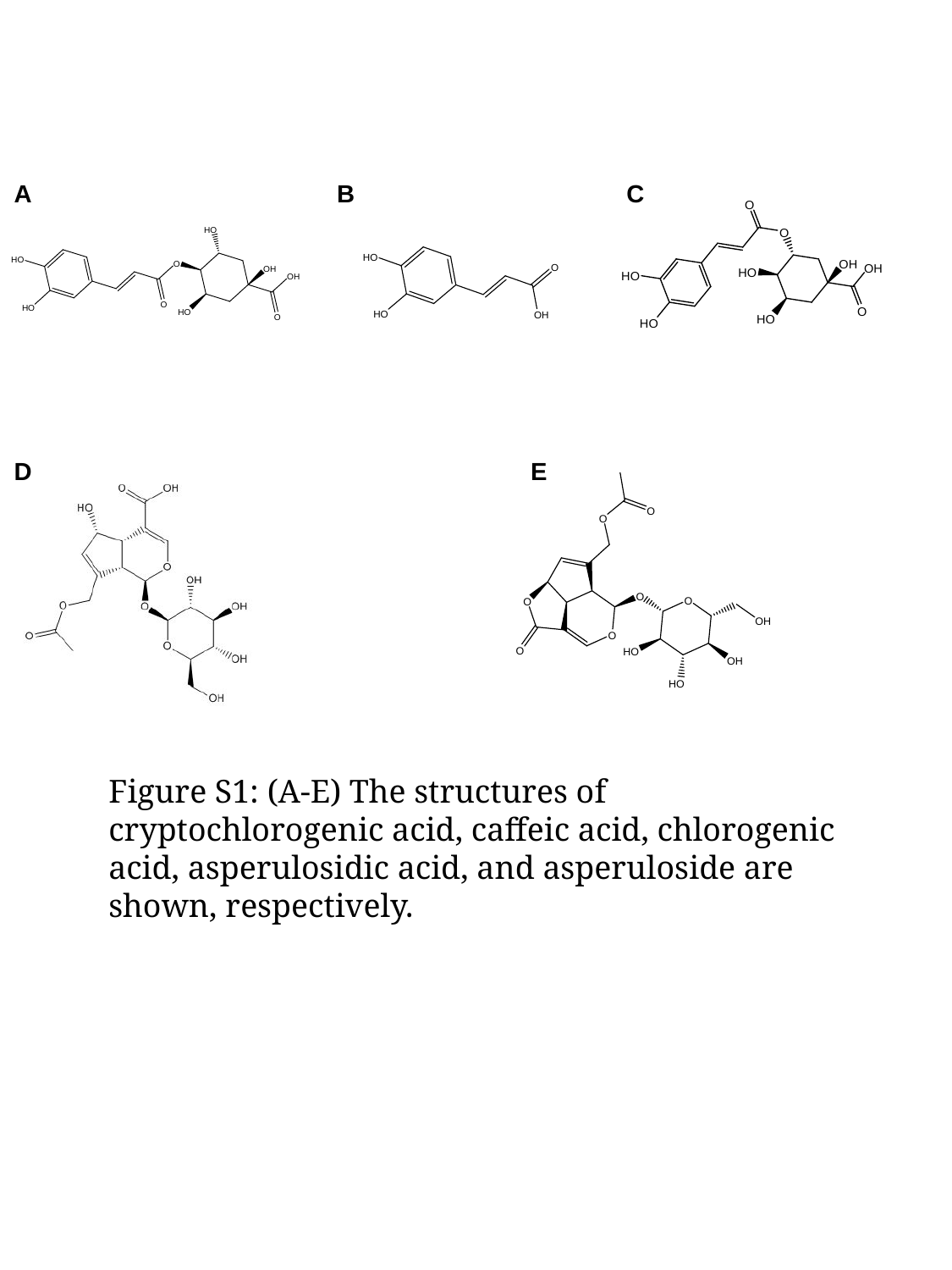

A
B
C
D
E
Figure S1: (A-E) The structures of cryptochlorogenic acid, caffeic acid, chlorogenic acid, asperulosidic acid, and asperuloside are shown, respectively.
