# Supplementary material for: Identification of Key Active Constituents in Eucommia ulmoides Oliv. Leaves Against Parkinson’s Disease and the Alleviative Effects via 4E-BP1 Up-Regulation
Source: Int J Mol Sci. 2025 Mar 19;26(6):2762. doi: 10.3390/ijms26062762 (PMC11943294; doi:10.3390/ijms26062762)
Supplement: Supplementary file 1 [file ijms-26-02762-s001.zip › Figure S2.pptx]

## Slide 1
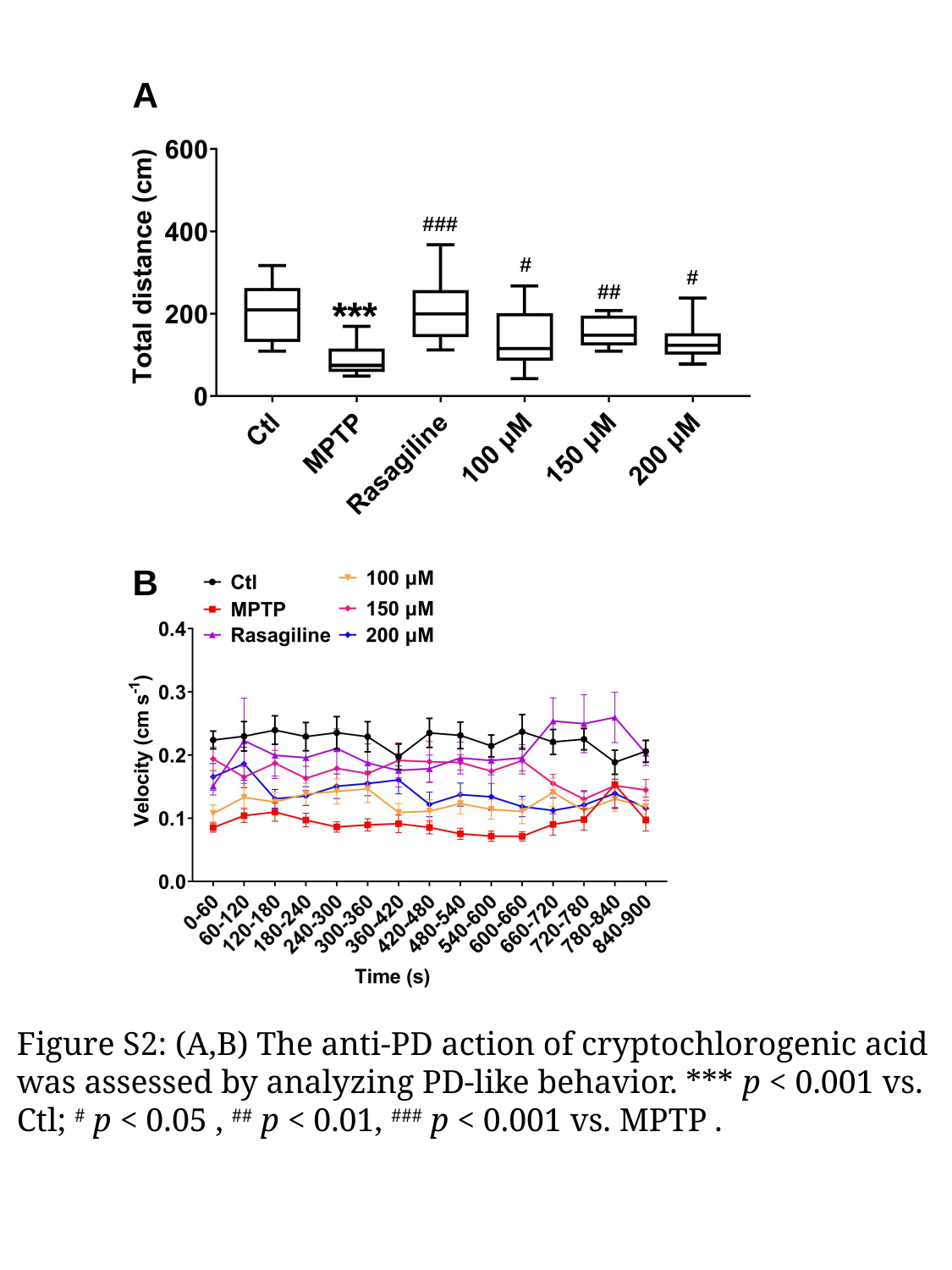

A
###
#
#
##
***
B
Figure S2: (A,B) The anti-PD action of cryptochlorogenic acid was assessed by analyzing PD-like behavior. *** p < 0.001 vs. Ctl; # p < 0.05 , ## p < 0.01, ### p < 0.001 vs. MPTP .
