# Supplementary material for: Identification of Key Active Constituents in Eucommia ulmoides Oliv. Leaves Against Parkinson’s Disease and the Alleviative Effects via 4E-BP1 Up-Regulation
Source: Int J Mol Sci. 2025 Mar 19;26(6):2762. doi: 10.3390/ijms26062762 (PMC11943294; doi:10.3390/ijms26062762)
Supplement: Supplementary file 1 [file ijms-26-02762-s001.zip › Figure S3.pptx]

## Slide 1
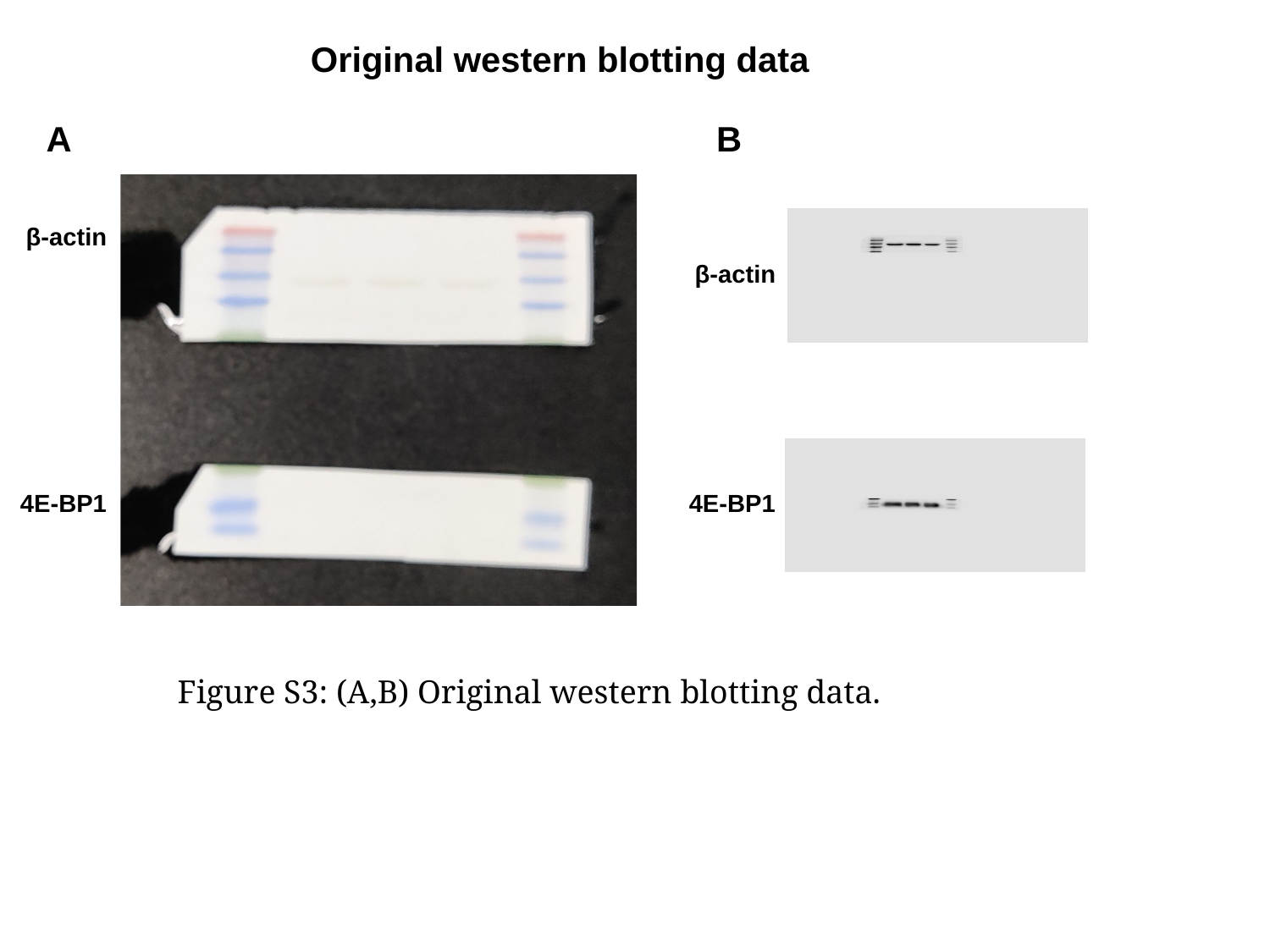

Original western blotting data
A
B
β-actin
β-actin
4E-BP1
4E-BP1
Figure S3: (A,B) Original western blotting data.
