# Supplementary material for: Identification of Key Active Constituents in Eucommia ulmoides Oliv. Leaves Against Parkinson’s Disease and the Alleviative Effects via 4E-BP1 Up-Regulation
Source: Int J Mol Sci. 2025 Mar 19;26(6):2762. doi: 10.3390/ijms26062762 (PMC11943294; doi:10.3390/ijms26062762)
Supplement: Supplementary file 1 [file ijms-26-02762-s001.zip › Table S1.pdf]

Table S1. The sequences of primer pairs used in RT-qPCR assay.

Gene	Sequence of the forward primer	Sequence of the reverse primer
rpl13a	TCTGGAGGACTGTAAGAGGTATGC	AGACGCACAATCTTGAGAGCAG
α-syn	ATGGATGTTTTTATGAAGGGGC	ACGCTGTCTTTGGTCTTGCT
atg7	AGAGTCCAGTCCGATGTC	GAAGTAACAGCCGAGACG
lc3b	CCTCCAACTCAACTCCAACC	GCCGTCTTCGTCTCTTTCC
p62	TTTGGCTCTTGTGAAGGATGAC	GAGGGCTAAAGTGAGGTGTAGTGA
