# Supplementary material for: Identification of Key Active Constituents in Eucommia ulmoides Oliv. Leaves Against Parkinson’s Disease and the Alleviative Effects via 4E-BP1 Up-Regulation
Source: Int J Mol Sci. 2025 Mar 19;26(6):2762. doi: 10.3390/ijms26062762 (PMC11943294; doi:10.3390/ijms26062762)
Supplement: Supplementary file 1 [file ijms-26-02762-s001.zip › Table S2.pdf]

Table S2. UPLC-Q-Exactive Orbitrap/MS identification of the active compounds from 30% EF extracts.

Compounds	t_R (min)	Ionization	Fragment	Precursor ion	Formula
Cryptochlorogenic acid	8.78	$[M-H]^-$	263, 191, 179, 173, 135, 93	353.0876	$C_{16}H_{18}O_9$
		$[M+H]^+$	263, 191, 179, 173, 135, 93	355.1021	
Caffeic acid	7.91	$[M-H]^-$	135, 107	179.0339	$C_9H_8O_4$
Chlorogenic acid	7.30	$[M-H]^-$	340, 191, 173, 161, 135, 127, 93, 85	353.0876	$C_{16}H_{18}O_9$
		$[M+H]^+$	337, 288, 181, 163, 145, 135, 117	355.1021	
Asperulosidic acid	7.04	$[M-H]^-$	251, 165, 121, 101, 89, 71, 59	431.1192	$C_{18}H_{24}O_{12}$
Asperuloside	9.63	$[M-H+HCOOH]^-$	413, 381, 251, 233, 191, 147, 119	459.1144	$C_{18}H_{22}O_{11}$
		$[M+Na]^+$	388, 275, 257, 215, 197, 187	437.1050	
Chlorogenic acid isomer	11.32	$[M-H]^-$	191, 161, 85	353.0876	$C_{16}H_{18}O_9$
